# The roles of abiotic factors, dispersal, and species interactions in structuring stream assemblages of black flies (Diptera: Simuliidae)

**DOI:** 10.1186/2046-9063-8-14

**Published:** 2012-06-28

**Authors:** John W McCreadie, Peter H Adler

**Affiliations:** 1 Department of Biological Sciences, University of South Alabama, Mobile, AL 36688, USA; 2 School of Agricultural, Forest & Environmental Sciences (Entomology), Clemson University, Clemson, SC 29634, USA

**Keywords:** Black flies, Community structure, Competition, Co-occurrence, Dispersal, Mantel tests, Null models, Simuliidae, Streams

## Abstract

**Background:**

The patterns and drivers of species assemblages represent the core of community ecology. We focus on the assemblages of a single family of ubiquitous lotic insects, the Simuliidae (black flies), of which the larvae play a critical role in resource turnover in steams. We use Mantel tests and null models to tease out the potential influence of abiotic stream conditions, species interactions, and dispersal on the assemblage patterns of larval black flies over two spatial scales (within and across ecoregions) and two seasons (spring and summer).

**Results:**

When stream sites were considered across ecoregions in the spring, stream conditions and dispersal were correlated significantly with species similarity; however, within ecoregions in the spring, dispersal was important only in the Piedmont and Sandhills and abiotic factors only in the Mountains. In contrast, results of the summer analyses within and across ecoregions were congruent; assemblage similarity was significantly correlated with stream conditions both across and within ecoregions. Null models suggested that patterns of species segregation in the spring were consistent with a community structured by competition, whereas patterns in the summer were consistent with species assemblages influenced by abiotic factors.

**Conclusions:**

Species composition of black flies at streams sites is correlated with dispersal factors and stream conditions, but results vary over spatial and temporal scales. Communities of black flies can be viewed within a metacommunity context; local assemblages are consistent with species sorting and mass effects. Given that black flies have a terrestrial stage, with females deciding where to place the eggs, a full understanding of the processes that determine local aquatic assemblages will require integration of the dynamics of the aquatic immature stages and the terrestrial adults.

## Background

Describing patterns of species assemblages and identifying the processes responsible for these patterns is central to community ecology [1]. Assemblages are driven directly and indirectly by abiotic and biotic factors and their interactions [2,3]. Theoretical developments of community structure, however, have outpaced empirical studies of species assemblages in many respects [4], prompting a need for investigations of different taxa across different ecosystems.

Assemblage patterns and their causative processes can vary with scale [5]. Species interactions (e.g., competition) and abiotic factors in streams likely filter species at local scales (i.e., the stream reach). At regional scales (i.e., across streams and ecoregions), changes in species composition reflect responses to environmental gradients and dispersal abilities [6]. Variation in species composition among local stream communities, therefore, results from the interplay among local abiotic and biotic conditions and large-scale factors (e.g., dispersal) operating over different spatial and temporal axes [7-9].

Taxa respond differently to abiotic and biotic factors and differ in their powers of dispersal. Lack of congruence among taxa, therefore, could obfuscate patterns of species assemblages. Consequently, we focus on one family of lotic insects, the Simuliidae. Larval black flies play an integral role in organic matter processing in streams [10], are taxonomically well known, and represent a predominantly single functional group of collector-filterers [11]. Larvae of many aquatic groups, however, can be identified only to genus, with broad taxonomic investigations leading to mixtures of different taxonomic levels. An advantage of the current study is the opportunity to examine patterns of community assemblages while minimizing the effects of taxonomy.

Larval black flies occupy habitats ranging from temporary trickles to large rivers, and often dominate the stream macroinvertebrate community [12]. Larvae adhere to solid substrates in streams and feed primarily by filtering or, less often, by scraping or preying on smaller organisms [13]. The females of most species require avian or mammalian blood for egg development and a sugar source (e.g., nectar) for energy, dispersing from their natal streams in search of hosts [11]. Many adult females can be difficult to identify to species; hence, determining fly-host relationships is impeded. After obtaining blood and sugar meals, the females return to streams to oviposit. Although rarely investigated, conditions in the terrestrial ecosystem, such as host availability and distances between streams, could affect species assemblages in lotic ecosystems.

We explore the patterns of species assemblages of larval black flies, using two separate, but complementary, approaches. We first examine the changes in species composition across abiotic gradients and correlate these changes with potential explanatory variables. We next examine assemblages for nonrandom patterns of species co-occurrences across stream sites. Co-occurrences typically are examined as species pairs common to one or more habitats [14,15]. Using two approaches to examine assemblage patterns provides the opportunity to isolate potential explanatory mechanisms. This dual approach is important if, as we suspect, community assemblages of black flies are regulated by multiple factors. Equally important is that many studies are built on the implicit assumption that the effects of multiple factors on community organization manifest across scales in a relatively consistent manner [4]. Using two approaches, employed over different spatial and temporal axes, has the potential to relax this assumption and at least partially untangle the complexity of processes underlying patterns of species assemblages.

We predict that 1) explanatory abiotic stream variables for assemblages of larval black flies should vary across ecoregions if ecoregions present different abiotic templates, 2) associations between spatial variables and species assemblages should decrease with decreasing distances (i.e., as dispersal limitations decrease), and 3) if co-occurrence patterns reflect changes in abiotic conditions, rather than biotic conditions (e.g., competition), these patterns should shift from segregation to aggregation or random associations as abiotic gradients decrease.

## Materials and methods

Details of the study area, sampling procedures, and stream measurements have been presented elsewhere [16] and are summarized briefly here.

### Study area

The study area included streams in western South Carolina, USA (33.25°—35.20°N, 80.68°—83.30°W). Rivers in South Carolina drain southeastward from the Appalachian Mountains to the Atlantic Ocean. The state grades into four ecoregions [17]: Blue Ridge Mountains, Piedmont, Sandhills, and Coastal Plain. To define more meaningful regional scales with regard to stream habitat, boundaries between ecoregions of upper South Carolina delineated by Myers [17] were modified as follows [16] : 1) Mountains: rugged to hilly terrain 240–1100 m above sea level (asl), with fine-loam, sandy-loam, and clay soils, 2) Piedmont: gentle rolling topography 70–400 m asl, with clay-textured soils, and 3) Sandhills: rolling to hilly landscape 70–200 m asl, with dry, sandy soils.

### Sampling regime

Study sites varied from rocky-bottomed mountain streams to sandy-bottomed blackwater streams. No standardized collecting procedure is relevant to all conditions, and timed collections have little meaning among sites that range from small torrents to meandering rivers. Accordingly, each site (i.e., each local assemblage) was sampled by walking transects from bank to bank while hand collecting larvae and pupae from all available substrates, with the intent of collecting a minimum of 30 specimens per site. We assumed that species in the sample from each site represent local occurrences [18,19]. Faunal lists of black flies that result from hand collections are the same as those produced using more quantitative and repeatable sampling units, such as artificial and natural substrates [20,21]. Only sites in which all specimens could be identified to species were used. This constraint produced data from 57 sites sampled from May to July 1992 (‘summer’ collections) and from 53 sites sampled from February to April 1993 (‘spring’ collections).

For each collection, we measured stream conductivity, depth, dissolved oxygen, pH, temperature, velocity, and width. We visually ranked riparian vegetation (open, brush, forest), dominant streambed-particle size (mud, sand, small stones, rubble, boulders, bedrock), and canopy cover (none, partial, complete). Discharge was calculated from stream depth, width, and velocity. Variables selected for measurement have been documented as useful predictors of insect distributions and diversity among stream reaches [18,22-24].

### Species identification

Larvae and pupae were fixed in three changes of acetic ethanol (1:3) and identified morphologically. Mid- to late instar larvae of taxa with isomorphic species were Feulgen-stained to examine their silk-gland polytene chromosomes [11]. To make cytospecific identifications, chromosome-banding patterns were compared with standard maps in the literature [25] or on file in the laboratory of PHA.

### Data analysis

**Figure 1:**
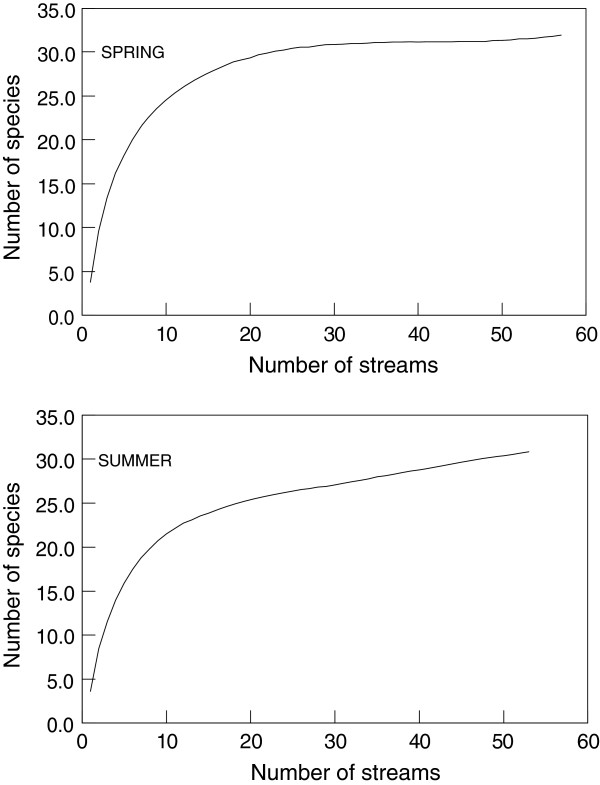
Species accumulation curves for larval black flies in spring and summer collections in South Carolina, USA.

All tests were considered significant at *P* < 0.05. Presence or absence of each species in each collection was expressed as binary data. Previous studies [26-28] have shown that binary data from single-point collections are robust for measuring faunal differences among streams. The intent of a multivariate analysis of community data is to separate structural patterns from noise [29]. Taxa most likely to be missed in a binary enumeration of a stream habitat are rare species. However, rare species often contribute to noisy data, and their inclusion can obscure the detection of informative correlative patterns [29]. We found no significant correlations between number of larvae collected and species richness for the spring (r = 0.094, p = 0.488) or summer (r = 0.211, p = 0.082). Species accumulation curves for both seasonal collections (Figure 1) suggested that our sampling procedure produced a reasonable representation of the regional species pool. Taken in total, we concluded that no excessive bias existed in our binary data.

Analyses were run separately for spring and summer collections. For each of the spring and summer collections, analyses between the species assemblages across sites and stream variables across sites were conducted using the BV-STEP routine [30], creating two matrices. The species (community) matrix was constructed using similarity among stream sites based on species composition. The ecological (abiotic) matrix was formed using similarity among sites based on stream variables. Thus, similarity among sites was measured using species data and abiotic data, each in a separate matrix. The species matrix was correlated with the ecological matrix, with the best subset of explanatory stream variables selected from the ecological matrix. Selection of the best explanatory variables was based on the strength of the correlation coefficient between the species matrix and every possible combination of stream variables from the ecological matrix [30]. Similarities for the species matrix were constructed using Sorensen distance, which performs well with binary data [31]. For the ecological matrix, each stream variable was log-transformed, if necessary, to stabilize the variance, and all stream variables were standardized (normalized) to a mean of zero and a variance of 1. Similarities in stream conditions between sites were calculated using simple Euclidean distances [29]. Once the best explanatory variables were selected, we determined the significance of the resulting correlation coefficient between the species matrix and subset of the best explanatory variables from the ecological matrix, using 999 random permutations. Routines were run using Primer v. 6© [30]. Analyses were conducted across ecoregions (i.e., all sites sampled) and within ecoregions.

To account for geographic distances between sites, which are typically interpreted as dispersal limitations [32], latitude and longitude coordinates were expressed as decimal degrees east (westernmost point = 0) and decimal degrees north (southernmost point = 0). Female black flies of some species can disperse up to hundreds of kilometers [13]; therefore, only simple first-order terms of Euclidean distance were considered to construct distance matrices. That is, we assumed that dispersal between sites was aerial and best approximated by linear distances, a reasonable assumption, given the life cycle of most black flies [11,13]. Distance matrices were then used in partial Mantel tests [33]. This test correlates a particular species matrix with the complementary distance matrix, while accounting for the effect of the stream conditions. In our case, stream conditions were the best subset of explaintory variables selected from the ecological matrix by the best BV-STEP routines. The partial Mantel test is essentially the multivariate equivalent of univariate partial correlations. The complementary analyses, that is the effect of the best subset of explanatory stream conditions, while accounting for the effect of distance, also were conducted. Partial Mantel tests were run using the PAST software package [34], with significance of each partial correlation based on 5000 (default) random permutations between the matrices.

Canonical correspondence analysis and redundancy analysis have been used to parcel out the variation due to effects of position, habitat, and position-habitat interactions [35]. However, the assumptions of these methods are rarely, if ever, satisfied by community (species) data [29,30]; we, therefore, elected to use the BV-STEP Routine and partial Mantel tests, which make few, if any, assumptions.

Null models were used to determine if patterns of species co-occurrences among stream reaches were nonrandom for the spring or summer collections. For any data set (matrix), observed co-occurrences less than expected by random—negative covariation of co-occurrences (i.e., species segregation)—are often interpreted as consistent with a community structured by competition [15,36,37]. Predation through a variety of mechanisms, such as direct food-chain predation, intraguild predation, and apparent competition, also could be expected to produce negative patterns of co-occurrence [38]. Negative covariation of co-occurrences also can emerge from differing responses of species to abiotic conditions [39,40]. Observed co-occurrences greater than expected by random—positive covariation (i.e., species aggregation)—could emerge as a result of species with similar abiotic requirements [15] or facilitation [41]. Habitats, such as streams, subject to a series of stochastic disturbances (e.g., spates) and recolonization events, might have co-occurrences no greater or less than expected by chance [14,15,42].

Each analysis entailed the construction of a presence/absence (1/0) data matrix with rows representing species and columns representing stream sites. Row margins represented frequency of occurrence for each species and column margins species richness at each site. These data sets are referred to as FULL to distinguish them from our data subsets described below. The algorithm used for randomizing matrices to create a test distribution of co-occurrence against which the observed test statistic (C-score) is compared fixes both the row margins (simulated species frequencies = observed species frequencies) and the column margins (simulated species richness at a site = observed species richness). This simulation has desirable Type I and Type II error properties [43]. Each randomization used the sequential swap to randomize data, and each analysis was based on 5000 randomizations, using the Ecosim© statistical package [37].

The C-score, measuring the number of checkerboard subunits, was used as our test statistic [44]. A checkerboard subunit occurs when species A is present at site 1 and absent at site 2, while the reverse is true for species B. In the co-occurrence matrix, a checkerboard subunit takes the form

0110

or

1001

The C-score is the average number of subunits between all possible species pairs. An observed C-score greater than expected by chance indicates an assemblage in which co-occurrences are less than expected by chance (negative co-variation or species segregation). An observed C-score less than expected by chance indicates an assemblage in which co-occurrences are greater than expected by chance (positive co-variation or species aggregation) [37].

To determine if patterns of co-occurrence were consistent with the expectations of a community structured, at least in part, by abiotic conditions, the complete data set (FULL) was subdivided into two groups: 1) streams with low variation in abiotic stream conditions (LOW data subset) and 2) streams with high variation in stream conditions (HIGH data subset). Grouping was determined by subjecting stream variables to a Principal Components Analysis (PCA). Although not appropriate for species data, PCA is appropriate for abiotic data [45]. Here, it is used as a simple, objective, and repeatable *a priori* method to define high and low variability in streams [28].

On an *a priori* basis, streams in which the first three Principal Components (PCs) were within 1.0 standard deviation of their means were considered to have low variation in abiotic conditions (LOW). The remaining streams were grouped as high-variation streams (HIGH). Null-model analyses were repeated for each of these two groups. If stream conditions were a major influence in structuring co-occurrence patterns, results of the analysis between the LOW data subset (‘control’) should differ from the results of the HIGH data subset (‘treatment’). The above PCA also was used to produce ordinations of stream conditions by ecoregion. Interpretation of the PCs was based on correlations between each PC and the original stream variables [16]. Differences in PCs across ecoregions for each season were determined using the nonparametric Multiple Analysis of Variance (MANOVA) with a Bonferroni correction of the p-value for the three pairwise comparisons among ecoregions [34].

In addition to the above null-model analyses, data from each ecoregion were analyzed for spring and summer collections, although sample sizes were too small to subdivide each of these data sets into LOW and HIGH subsets. For each analysis, in addition to the p-value, effect size was calculated as (C_obs_ – C_m_)/SD, where C_obs_ = observed C-score, C_m_ = mean C-score of the test distribution, and SD = standard deviation of the test distribution [40].

## Results

The mean (±S.E.) number of larvae collected per site did not differ significantly (t = −0.60, *P* = 0.552, df = 95) between the spring (116.4 ± 13.40) and summer (125.8 ± 8.35). We identified 37 species, 28 in the spring and 25 in the summer, with 16 species common to both seasons.

For spring collections, PC_1 _was largely a measure of stream size and PC_2 _a broad measure of flow (Table 1). PC_3 _was a measure of bankside vegetation. Similar to the spring collections, PC_1 _and PC_2 _for the summer collections were largely measures of stream size; PC_3 _was related to changes in water chemistry (pH, conductivity). The nonparametric MANOVA with a Bonferroni correction for number of comparisons indicated that PCs differed significantly (*P* < 0.001) among the three ecoregions in both the spring and summer. We, therefore, concluded that each ecoregion presented a distinct abiotic environment, maintained between seasons.

**Table 1:** PCA and subsequent correlation analysis between stream variables and derived principal components (PCs)

**Stream variables**				**Principal components **^**2**^		
	**Min.**	**Max.**	**Mean ( ± SE)**	**PC**_**1**_	**PC**_**2**_	**PC**_**3**_
**Spring**						
Temperature (°C)	7.0	16.0	11.1 ± 0.30	-0.505 **	-0.363 *	-0.118
pH	5.6	7.5	6.5 ± 0.05	0.554 **	-0.383 *	0.325
% Dissolved Oxygen	93.2	115.4	102 ± 0.66	-0.131	-0.710 **	0.130
Conductivity (°S cm^-1^, 25°C)	16	152	40 ± 3.2	0.483 **	-0.073	0.377 **
Depth (m)	0.08	1.88	0.40 ± 0.047	0.783 **	0.258	-0.250
Velocity (m/s)	0.09	0.87	0.40 ± 0.024	-0.020	-0.816 **	-0.139
Width (m)	0.5	20.0	6.6 ± 0.67	0.807 **	-0.005	-0.272
Discharge (m^3^/s)	0.01	17.9	1.5 ± 0.40	0.861 **	-0.089	-0.280
Seston (mg/L)	2.0	74.0	14.0 ± 0.30	0.144	-0.623 **	0.151
Streambed-particle size ^**1**^	1.0	6.0	2.0 ± 0.26	-0.308	-0.788 **	-0.123
Riparian vegetation ^**1**^	1.0	3.0	2.1 ± 0.14	-0.347 *	-0.087	-0.736 **
Canopy cover ^**1**^	1.0	3.0	1.6 ± 0.09	-0.418 *	0.151	-0.655 **
% variance explained in PCA						
Proportion				24.9	21.8	15.0
Cumulative				24.9	46.8	61.7
**Summer**						
Temperature (° C)	13.0	29.0	20.0 ± 0.49	0.514 **	-0.560 **	0.054
pH	5.80	7.9	7.1 ± 0.06	0.085	0.239	0.782 **
% Dissolved Oxygen	78.8	106.7	93.25 ± 0.93	-0.021	0.650 **	-0.433 *
Conductivity (°S cm^-1^, 25°C)	10	235	73 ± 7.46	0.303	-0.137	0.897 **
Depth (m)	0.03	0.73	0.18 ± 0.029	0.873 **	0.003	-0.124
Velocity (m/s)	0.02	0.67	0.31 ± 0.018	0.424 *	0.616 **	-0.296
Width (m)	0.30	36.00	7.5 ± 1.20	0.871**	0.162	0.219
Discharge (m^3^/s)	0.01	9.38	0.73 ± 0.209	0.889 **	0.236	0.019
Seston (mg/L)	2.8	50.0	14.6 ± 0.49	0.237	-0.079	0.091
Streambed-particle size ^**1**^	1.0	6	2.0 ± 0.24	-0.124	0.892 **	-0.098
Riparian vegetation ^**1**^	1.0	3	2.3 ± 0.13	-0.121	0.386 *	0.076
Canopy cover ^**1**^	1.0	3.	2.0 ± 0.15	-0.473 **	0.220	0.056
% variance explained in PCA						
Proportion				26.4	20.3	15.8
Cumulative				26.4	46.8	62.6

^**1 **^Dominant streambed-particle size (mud = 1, sand = 2, small stones = 3, rubble = 4, boulders = 5, bedrock = 6), riparian vegetation (open = 1, brush = 2, forest = 3) and canopy cover (none = 1, partial = 2, complete = 3) are ranked variables, with medians presented.

^**2 **^Significant correlation at * *P* < 0.01, ** *P* < 0.001.

When all sites were considered (across-ecoregion scale), both stream variables and distances between sites in the spring collection were correlated significantly with species similarity, as indicated by the partial Mantel tests (Table 2). Significant stream variables selected by the BV-STEP routine were pH, seston, streambed, and velocity. However, when the analysis was delineated by ecoregion, results differed. In the Mountain ecoregion, only stream variables were correlated significantly with species similarity. The only significant stream explanatory variable in common between these two analyses (i.e., All sites, Mountain sites) was velocity (Table 2). For both the Piedmont and Sandhills ecoregions, only distance between stream sites was correlated significantly with the species matrix. Hence, our prediction of significant correlations between distance among sites and species similarity of species assemblages was highly supported in the spring collections. The partial Mantel tests for stream variables for the Piedmont and Sandhills ecoregions were not significant. Stream variables selected by BV-STEP, therefore, have little meaning.

**Table 2:** BV-STEP routine and partial Mantel tests between species resemblance matrices, stream variables and stream position

**BV-STEP routine**			**Partial mantel correlation coefficients**	
**Best explanatory variables**		**Correlation**^**1**^**coefficient**	**Stream variable**^**1**^	**Position**^**2**^
**Spring**				
All ecoregions	velocity pH, seston, bed	0.4961***	0.3000***	0.3795 ***
Mountain	velocity, O_2_, depth, canopy	0.3584 ***	0.3588 ***	- 0.0270
Piedmont	velocity,temperature discharge conductivity	0.5127 **	0.0478	0.6085 ***
Sandhills	temperature, width, discharge	0.1632 *	0.1532	0.2864 **
**Summer**^**4**^				
All ecoregions	temperature,conductivity bed	0.5631***	0.4965***	0.1069
Mountain	temperature, width, bed	0.5337***	0.5071***	0.0391
Piedmont	temperature, bed	0.4341***	0.4318***	0.0268

^**1**^ * *P* < 0.05, ** *P* < 0.01, *** *P* < 0.001.

^**2**^ Partial correlation of the species matrix with stream variables, accounting for the effect of distance.

^**3 **^Partial correlation of the species matrix with distance variables, accounting for the effect of stream variables**.**

^**4 **^Due to the low number of sites (n = 6), simple and partial Mantel tests were not calculated for Sandhills collections during the summer**.**

In contrast to the spring collections, results of the summer analyses were highly similar. Assemblage similarity was significantly correlated only with stream conditions and these correlations were significant across and within ecoregions. Temperature and streambed were selected as significant explanatory variables in all cases. Analysis of the Sandhills within ecoregion was not conducted; too few collections (n = 6) were available to calculate meaningful tests. Thus, our prediction that explanatory abiotic stream variables for local assemblages of larval black flies should vary among ecoregions was not supported, at least for the summer collections.

**Figure 2:**
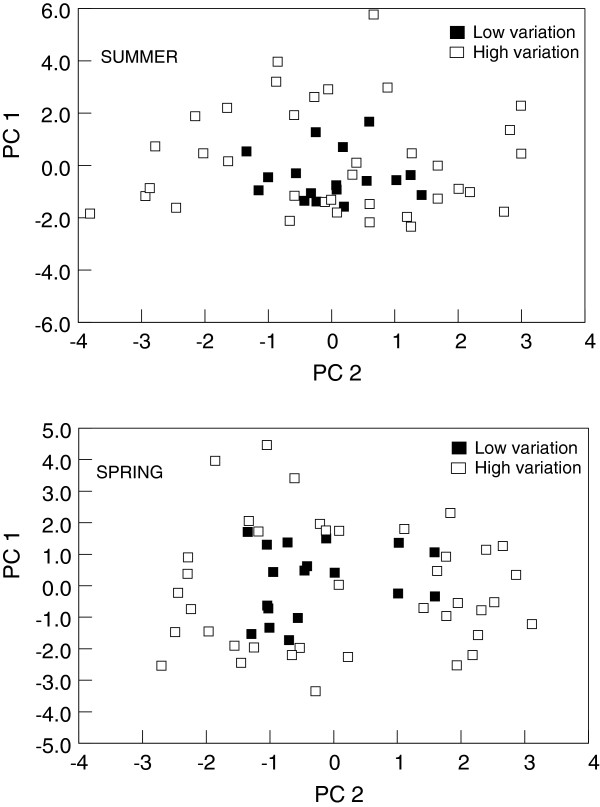
Ordination of the first two PCs of stream variables for spring and summer collections of larval black flies in South Carolina, USA.

Ordination of streams on PC_1 _and PC_2 _(Figure 2) showed that the relative variation of stream parameters among streams in the LOW group was lower than the relative variation in the HIGH group. When the FULL data set was examined for the spring collections, the number of checkerboard units was greater than expected by chance (Table 3), indicating that species showed significant segregation among sites. These results were mirrored by the null-model analyses of both the HIGH and LOW data subsets. The agreement of the LOW data subset (low variation in stream conditions) with the HIGH subset (high variation in stream conditions) indicates stream conditions were not a major determinant of species segregation. This conclusion is supported by the Mantel tests that showed distance among sites was more often significantly correlated with community similarity than with stream conditions (Table 2). For the summer collections, the number of checkerboard units for both the FULL and HIGH data sets were greater than expected by chance, indicating that species co-occurrences were less than expected by chance (i.e., species showed significant segregation). In contrast, when we partially controlled for abotic conditions (i.e., defined a relatively homogenous group of streams; LOW data subset), no significant segregation was found, suggesting that abiotic factors influenced the segregation of species in the FULL and HIGH data sets. This interpretation agrees with the Mantel tests (Table 2) for the summer collections. Accordingly, null model analyses and Mantel tests, taken together, support our third prediction that if differences in species assemblages reflect changes in abiotic conditions, rather than biotic conditions (e.g., competition), patterns should shift from segregation to aggregation or random associations as abiotic gradients decrease.

**Table 3:** Results of null-model analyses of species co-occurrences of larval black flies

**Data set**	**Season**	
	**Spring**	**Summer**
**FULL data set**		
Observed C-score	33.47	30.56
Mean C-score ^1^	31.99	29.13
p ^2^	<0.0001	<0.0001
effect size ^3^	5.15	4.25
n	57	53
**HIGH subset**		
Observed C-score	15.78	17.08
Mean C-score	15.39	16.40
p	<0.0204	<0.0001
effect size	2.24	4.10
n	39	36
**LOW subset**		
Observed C-score	5.11	5.42
Mean C-score	4.69	5.40
p	0.0002	0.5890
effect size	4.26	0.11
n	18	17
**MOUNTAIN ECOREGION**		
Observed C-score	6.39	6.72
Mean C-score	6.41	2.62
p	0.5280	>0.0001
effect size	-0.09	7.25
n	18	17
**PIEDMONT ECOREGION**		
Observed C-score	4.32	13.53
Mean C-score	3.86	13.08
p	0.0110	0.0790
effect size	2.95	1.49
n	16	31
**SANDHILLS ECOREGION**^**4**^		
Observed C-score	7.06	-
Mean C-score	7.00	-
p	0.2580	-
effect size	0.63	-
n	19	-

^**1 **^Mean C-scores are the means from the test distribution generated using the randomization algorithms.

^**2 **^p is the probability of a C-score > observed C-score under a random model

^**3 **^effect size was calculated as (C_obs_ – C_m_)/SD, where C_obs_ = observed C-score, C_m_ = mean C-score of the test distribution, and SD = standard deviation of the test distribution.

^**4 **^Due to the low number of sites (n = 6), null-model analysis was not performed for Sandhills collections in the summer.

Overall, our results indicated that both the distances between sites (dispersal factors) and the site conditions were significantly correlated with community composition of larval simuliids. The relative importance of these factors, however, varied along spatial and temporal axes. Species segregation during the spring was consistent with a community influenced by competition (biotic factors); species segregation during the summer appeared to be the result of stream conditions.

## Discussion

Assemblages of black flies in flowing waters of the southeastern United States are dynamic, changing with season across a spatial patchwork of environmental factors. Distributions of the component species are predictable over multiple scales from an in-stream substrate [22,46], across streams, to ecoregion, even in the absence of physical barriers to dispersal [16]. The factors responsible for distributions at smaller scales (within a stream reach) can be manipulated experimentally and, therefore, are reasonably well understood for their role in driving assemblage patterns. Our study addresses the more elusive drivers of species assemblages at larger scales—within and across ecoregions. Accordingly, we demonstrated that shifts in abiotic conditions across ecoregions were met with corresponding shifts in local species assemblages. Other studies also have found abiotic gradients in freshwater habitats across ecoregions, with accompanying differences in faunal characteristics [26,47-49].

Diamond [36] used presence/absence data from the bird community of the Bismark Archipelago to infer a number of assemblage rules, starting a debate that has lasted more than 35 years. One sticking point has been whether null-model approaches can be used to infer causal mechanisms of species distributions over large scales. Nonetheless, the use of well-behaved statistical null models by a number of authors has lead to a deeper understanding of the processes structuring communities [28,40,43,50]. Inference is typically made by proposing expected outcomes, given that the mechanism under consideration has no influence on distribution, and comparing these outcomes to observational data. This approach is essential, as traditional manipulative experiments at spatial scales required to document regional patterns and processes are often not possible, or in some cases, even ethical [51]. Furthermore, experiments at the local level (e.g., stream reach) might not accurately reflect the nature of species patterns at the regional level (e.g., among streams) [52].

We examined patterns of species co-occurrences at regional scales, with the idea that patterns of co-occurrence across habitats reflect both community structure and the mechanisms responsible for the structure. Null-model analyses reveal a strong pattern of negative co-occurrences for the spring and summer FULL data sets. In most communities, species co-occurrences are fewer than expected by chance, a major exception being some invertebrate assemblages [39]. These segregated patterns of co-occurrence usually result either from ecological checkerboards (e.g., competitively structured assemblages) [15,36,43,53] or habitat checkerboards (e.g., assemblages structured by abiotic variables) [28,39,40]. For a given assemblage, the difficulty has been in determining the dominant causal mechanism [54]. To provide insights into potential causal mechanisms, we controlled for abiotic factors and then re-examined the patterns of co-occurrence. By dividing streams into two groups for which variation in their conditions was either low or high, we controlled, at least partially, for the influence of abiotic conditions on species distributions.

When we controlled for the influence of abiotic conditions (LOW), species during the spring remained strongly segregated, consistent with a community structured by competition. Predation also could produce negative patterns of co-occurrence [38]. However, given that we considered only a single guild (filter-feeding) of closely related species (79% in the genus *Simulium*), competition would seem a likely explanation. Considerable evidence shows competitive displacement between species of black flies and between black flies and other insects at local scales, with the limiting resource being suitable substrates for attachment and locations on those substrates for optimal food delivery [55-57]. Although a variety of organisms feed on larval black flies [58,59], their influence on displacement is poorly understood. Black flies might be able to minimize the effects of predation by a preference for high stream currents [58]. Although predation cannot be dismissed, our study suggests that competition in local stream habitats can scale up to produce competitively driven co-occurrence patterns at regional scales. Recent evidence suggests that local competitive interactions among birds can scale up to influence broad-scale distributions [3].

In contrast to the spring, the highly segregated community in the FULL and HIGH data sets shifted to an unsegregated community in the LOW data set during the summer, suggesting that stream conditions were responsible, consistent with a community structured largely by abotic factors [28]. Our data suggest that the causal mechanisms accounting, at least partly, for the patterns of species co-occurrences can shift seasonally from biotic to abiotic factors. Of particular interest with regard to biotic factors is that scaling-up from the local assemblage to the regional level is seasonally dependent. The finding that causal mechanisms of species co-occurrences can shift seasonally from biotic to abiotic drivers reinforces the view that a comprehensive understanding of community ecology requires appreciation of temporal, as well as spatial, scales [60]. The reasons behind seasonal shifts in causal mechanisms of the pattern of species distributions are not known. However, species composition of black flies in temperate regions changes through the year, with richness peaking in the spring and declining through the summer and fall [11]. As the number of species increases, competition would be expected to play a greater role in determining species distributions.

Mantel tests also show seasonal shifts between correlates of community structure. Given the results of our analyses, we argue that distances between sites (dispersal) are at least as important as local stream conditions during the spring. In contrast, during the summer, only local stream conditions are significantly associated with changes in species composition across sites. Dispersal abilities of the females, thus, might have a seasonal or climatic, as well as a taxonomic or phylogenetic, component. The females of most species of black flies typically disperse from their natal streams in search of hosts [13]. Species in the genus *Simulium*, especially those active in warmer months (e.g., summer) and hotter climates (e.g., Africa), generally disperse greater distances than spring-active species [13]. Thus, the importance of distance between sites could be less important in the summer than in the spring, which might account for the seasonal differences in the results of our Mantel tests.

Integrating results of null-model analyses and Mantel tests can be accomplished within a metacommunity framework. A metacommunity may be viewed as a group of communities (assemblages) of potentially interacting species interconnected by dispersal [60]. Given that black flies leave their natal waters in search of blood meals and can disperse many kilometers [13], the idea of a metacommunity framework in which to conceptualize simuliid assemblages in the lotic environment is appealing. Although studies in metacommunity dynamics often seek to determine if particular communities are structured by factors that are either stochastic (e.g., neutral models) or deterministic (e.g., niche models), such approaches are not useful; numerous factors likely operate simultaneously in a particular community [61]. Given this caveat, our empirical results are consistent with two conceptual aspects of metacommunity dynamics—species sorting and mass effects.

Species sorting is a niche-based view of the community in which species occur in local habitats where abiotic and biotic conditions are favorable [62]. Mass effects consider not only local conditions but also how dispersal can affect local community dynamics [63]. A strong association of local communities with local stream conditions would indicate species sorting. A significant association between site location and community composition would suggest that dispersal among sites (mass effects) also can influence local communities.

Both species sorting and mass effects could be expected to operate partly through oviposition behavior [28]. Although rarely considered in detail, the idea is simple. A stream insect is often in a particular location because the adult female placed the egg in that location. Although larval black flies drift within a stream reach, no evidence has suggested that drift can influence the composition of local species assemblages [13]. In other words, a critical initial filter that determines the presence of species at a stream site is the oviposition decision of the female. Understanding local stream assemblages, therefore, involves a strong behavioral component. The idea that oviposition behavior is a key factor in stream assemblages dovetails with the idea of cross-ecosystem interactions [64]. For example, variation in stream conditions might have little influence on differences in local larval assemblages if a significant number of adult females failed to find appropriate terrestrial hosts. Thus, changes in bird or mammal abundances or distributions could influence local assemblages of aquatic insects see [65].

We know little about the effects of oviposition preferences of black flies (or most other stream insects) on the structure of stream communities. What is apparent is that oviposition behaviors differ among species. Females of some species, for example, deposit their eggs on vegetation, whereas others drop them into the water [13], and some species return to the natal waters to oviposit, whereas others do not [66,67]. Furthermore, the distribution of blood hosts might influence dispersal patterns and the distances traveled. We predict that generalist blood feeders have more general distributions than do host specialists. The occurrence of the immature stages of *S. annulus* at lake outflows in Sweden, for instance, has been related to the presence of loons (*Gavia*), preferred hosts of the female flies [68].

An understanding of community assemblages of larval black flies has been building over the past 30 years. Patterns at local and regional scales, and their changes across seasons, are reasonably well known, with a good degree of predictability [16]. Causal factors underlying these patterns become more elusive as scale increases, although we have shown that null models and Mantel tests can provide insights.

## Conclusions

Our study focused on the drivers of community structure for local species assemblages. By examining patterns of species distribution, with the assumption that these patterns reflect both community structure and the mechanisms driving the structure, we suggest that black fly communities can be viewed within a metacommunity framework. Accordingly, local species assemblages were consistent with species sorting and mass effects. However, the processes that determine local stream assemblages are both complex and scale dependent. This statement is hardly surprising. However, given that studies are often conducted over small spatial scales and short time periods, it explains why different conclusions are reached about the processes structuring lotic communities. For example, in our study, species co-occurrences of black flies among ecoregions were consistent with assemblages influenced by competitive interactions during the spring. But, co-occurrence patterns in the summer were consistent with assemblages structured by abiotic factors. If our study had been conducted over a single season, different conclusions would have been reached about the importance of stream conditions and species interactions in structuring species co-occurrences. For stream insects with strong interactions between the aquatic and terrestrial environments, an understanding of the processes that determine local stream assemblages will require integration of the organismal dynamics in both systems.

## Competing interests

The authors declare they have no competing interests.

## Authors’ contributions

JWM, PHA contributed equally to the paper. Both authors read and approved the final manuscript.
